# Factors affecting production of *Scopulariopsis brevicaulis* spores for use in self-healing concrete

**DOI:** 10.1007/s00449-025-03189-3

**Published:** 2025-06-25

**Authors:** Ahsanul Kabir Sumon, Lu-Kwang Ju

**Affiliations:** 1 Department of Chemical, Biomolecular, and Corrosion Engineering, The University of Akron, Akron, OH 44325 USA; 2 Present Address: Tremco CPG Inc., 3735 Green Road, Beachwood, OH 44122 USA

**Keywords:** *Scopulariopsis brevicaulis*, Solid-state fermentation, Soy hull, Spore

## Abstract

Concrete durability is compromised by its susceptibility to cracking, necessitating innovative solutions like self-healing concrete (SHC). *Scopulariopsis brevicaulis* is capable of biomineralization and its spores were found to hold high potential for use in SHC. Realizing this potential requires clean and effective production of *S. brevicaulis* spores, which remains unexplored. Here the factors and processes conducive to high productivity of *S. brevicaulis* spores were investigated. Suitability of cheap, renewable soy-based substrates: soy molasses (SM), soy hull (SH), and soy flour (SF) were first evaluated, and SH was found suitable. The comparison of SH-based solid-state fermentation (SSF) with submerged fermentation (SmF) revealed SSF’s superiority, producing spores earlier and with a more than 4.5-fold higher rate. Further study of SSF parameters, including initial spore inoculum, moisture, SH particle size, sugar supplementation, N-source supplementation, pH, salt addition, light (vs. dark) condition, and occasional mixing/shaking plus water addition, highlighted conditions that significantly boost spore production. Optimal moisture content (60–67%) and elevated medium pH (10–11) and salt addition (15 g/L NaCl) were key to enhancing yield, the latter likely induced stress-driven sporulation. Using larger SH particles (> 850 µm) also proved beneficial, improving oxygen transfer. Electron microscopy confirmed the effective attachment and penetration of spore chains into SH particles. This work significantly improved the technical and economic feasibility of producing *S. brevicaulis* spores for industrial SHC development.

## Introduction

Concrete, made with cement as the key ingredient, is the second most widely used material after water [1]. In 2022, more than 4.1 billion tons (4.4 billion tons in 2021) of new cement were produced [2]. It is estimated that production of each ton of cement consumes 3.4 GJ of thermal energy (in dry process) and 110 kWh of electrical energy and it releases 0.73–0.99 tons of CO_2_ [3]. Accordingly, it is reported that 12–15% of the total industrial energy use is spent for making cement [4], and 7–8% of all anthropogenic CO_2_ emissions is due to concrete production [5]. Clearly, reducing concrete production by prolonging concrete service life can reduce energy consumption and CO_2_ emission and significantly improve the sustainability and environmental friendliness of human activities.

Concrete is strong but can crack for various reasons [6], which weakens the concrete, allows permeation of water, air, salt, and other chemicals, exposes the reinforcing steel to corrosive factors, creates undesirable appearance, and causes safety concerns. Introducing self-repairing mechanisms to seal the cracks as they appear can substantially reduce these problems. Biomineralization, where microorganisms form solid deposits under suitable conditions, is a feasible mechanism for development of self-healing concrete (SHC). Attempts have been made with live cultures [7, 8] and, much more extensively, bacterial spores [9–11]. But, producing bacterial spores is expensive, particularly because of the small bacterial spore size (e.g., < 1 μm) and the large medium volume to process for collecting a low concentration of spores, and the cost of cultivation medium. These factors led to high costs estimated for producing SHC based on bacteria spores, per m^3^ concrete: €5760 based on *Bacillus sphaericus* spores and €714 based on non-axenic mixed culture [12]. More recently, fungal spores have been proposed as an alternative for biorepair of cementitious materials, objects, and structures [13–17]. The fungal spores are much larger, 2–6 μm [18–20], and easier to collect than bacterial endospores. Fungi are known for producing extracellular enzymes [21–23], enabling growth by solid-state fermentation (SSF) on cheap or waste solid substrates with no or minimal free water. Spore collection from SSF avoids processing large liquid volumes with energy-consuming centrifugation. Accordingly, SSF offers a cleaner, low-cost, energy-efficient, and low-waste-generating way of producing fungal spores, compared to bacterial spores.

SSF is a versatile biotechnological process with a wide range of applications across various industries, including the production of industrial enzymes [24], functional foods, medicinal ingredients [25], composite materials [26], packaging and furniture [27], and construction and insulation materials [28]. SSF has also been used to produce fungal spores as biopesticides to control arthropods [29] and plant parasitic nematodes [30, 31]. SSF with rice husk as substrate was studied for producing spores of *Beauveria bassiana*, a pathogenic fungus to pests such as termites, thrips, whiteflies, aphids, and beetles [32]. The spores of the entomopathogenic fungus *Verticillium lecanii* were produced by SSF on sugarcane bagasse [33]. *Coniothyrium minitans* can control the crop diseases caused by *Sclerotinia*, and production of *C. minitans* spores was studied by SSF with wheat bran [34] or oats [35] as the solid substrate. Spore production of *Clonostachys rosea*, a biopesticide for the gray mold of strawberry, was observed in SSF with white rice grain [36]. SSF was also studied for spore production of several *Trichoderma* species using different solid substrates [37–39].

The fungus *Scopulariopsis brevicaulis* has been reported for making products such as fructooligosaccharides [40], keratinase [41], potential anti-cancer drugs [42], and chitin deacetylase [43]. Most recently, the use of *S. brevicaulis* spores for SHC development has been proposed, after its selection from a screening study due to its higher tolerance to the elevated pH and temperature conditions associated with cement hydration, as well as its superior ability to produce calcite [44, 45]. The global demand for concrete is 30 billion tons annually [46]. If 10% of the new concrete adopts SHC with 0.1% spores, 3 million tons of spores need to be produced each year. To meet this huge demand, the spores must be produced in an environmentally friendly manner with minimal waste. In this work, the cost effective, environmentally friendly, and cleaner method of producing *S. brevicaulis* spores by SSF was studied, following a comparison with production by submerged fermentation (SmF). Soy materials were studied as substrate, because soybean is a major crop globally and the second most produced crop in US [47] and because soybean has the highest protein content [48] so the use of soy materials may avoid additional N-source requirement for spore production. Three soy-derived materials were evaluated as substrate: soy molasses (SM), soy hull (SH) and soy flour (SF). SM and SH are waste/byproduct from soy processing, while SF is significantly more costly.

Fungi can sporulate when encountering harsh or growth-limiting conditions, often needing certain maturation time before sporulation [49]. Depending on the fungi, sporulation may be triggered/affected by several factors: light [50], temperature, humidity [51], aeration [52], pH [53], injury to the culture [54], nutrient type and composition [55], and starvation [56]. These factors were investigated in this study. In addition, the effect of soy hull piece size used as the solid substrate was examined, based on the hypothesis that larger pieces, by increasing SSF bed porosity, could enhance spore productivity. The impact of salt (NaCl) addition on spore production was also studied, under the assumption that the resulting osmotic stress might influence fungal sporulation. This is the first ever study on SSF cultivation of *S. brevicaulis* and on its spore production. The study offers a systematic evaluation of various factors on fungal spore productivity in SSF and concludes on the best substrate, condition, and parameters that gave maximum *S. brevicaulis* spore production per g substrate.

## Materials and methods

### Microorganism and materials

*S. brevicaulis* NRRL 1100 was obtained from the Agricultural Research Service (ARS) culture collection of the US Department of Agriculture. The culture was maintained on plates of 40 g/L potato dextrose agar (PDA, Sigma Aldrich) containing 4.1 g/L potato infusion (prepared from 205 g potatoes), 20.5 g/L dextrose, and 15.4 g/L agar. SH, SM, and SF were provided by the Archer Daniels Midland Company (Decatur, IL, USA). SM generally has more than 50% soluble solids, which contains about 60% carbohydrates, 10% nitrogenous substances, 20% lipids, and 10% minerals [57]. SH has about 7–8% moisture, 85.7% carbohydrates, 9% protein, 4.3% ash, and 1% lipids [58]. SF has about 52% protein, 33.3% carbohydrate, 5.2% moisture, 6.3% ash, and 3.2% fat [59].

### Study design

The study examined spore production of *S. Brevicaulis* in three types of cultivation systems: agar plates, SmF, and SSF, including investigation of all potential sporulation-triggering factors identified from literature survey. Agar plates were first used to evaluate the suitability of SH, SM and SF as substrate and the effects of light, pH and salt addition. The experiments were then made to compare spore production in SmF and SSF, using SH as substrate. SSF was chosen for further study, in two phases, to identify important factors that increase spore productivity and observe the time profiles of spore production (first phase) and to examine the factors for reproducibility with triplicate SSF flasks harvested after 14 days (second phase).

### Agar-plate tests on soy materials as substrate and effects of high pH, salt, and darkness on sporulation

The tests were all done at room temperature for 14 days of fungal growth and sporulation, with 9-cm Petri dishes that contained about 25 mL of different materials. As shown in Table 1, nine systems were tested, each with four replicates. One was the control system with *S. brevicaulis* growing on 40 g/L PDA at pH 7. In three systems, the PDA was replaced by 15 g/L agar plus 40 g/L of SH, SF, or SM, to evaluate the suitability of these soy materials. Two other PDA systems were made with 15 g/L NaCl and pH 10, respectively, to test the effects of high pH and salt on sporulation. The above six systems were kept under ambient light. The remaining three systems, made with SH, SM, or pH 10 PDA, were kept in dark to evaluate the effect of light conditions. The spore productivity of these systems was reported as the number of spores produced per cm^2^ of plate surface area. For each system, the average and standard deviation from 12 numbers (4 plates × 3 counts from each plate) are reported.

**Table 1:** Systems tested with agar plates

System	Organic substrate	Additional NaCl	Initial pH	Light condition
1 (control)	40 g/L Potato Dextrose Agar	No	7	Light
2	40 g/L SH, 15 g/L agar	No	7	Light
3	40 g/L SF, 15 g/L agar	No	7	Light
4	40 g/L SM, 15 g/L agar	No	7	Light
5	40 g/L Potato Dextrose Agar	15 g/L	7	Light
6	40 g/L Potato Dextrose Agar	No	10	Light
7	40 g/L SH, 15 g/L agar	No	7	Dark
8	40 g/L SM, 15 g/L agar	No	7	Dark
9	40 g/L Potato Dextrose Agar	No	10	Dark

### SmF vs. SSF

The SmF experiments were made in triplicate 500 mL Erlenmeyer flasks containing 80 mL water, 10 g SH, 2 g glucose, 0.2 g (NH_4_)_2_SO_4_ (Fisher Scientific A702), 0.1 g K_2_HPO_4_ (Fisher Scientific P290), 0.025 g CaCl_2_⋅2H_2_O (Fisher Scientific C793), 0.025 g MgCl_2_∙6H_2_O (Sigma Aldrich M8266), and 0.02 g FeSO_4_∙7H_2_O (Fisher Scientific I146). The mixture pH was about 5.5. The flasks were covered with cheesecloth-wrapped cotton, autoclaved at 121 °C for 20 min, and, after being cooled to room temperature, inoculated with 2.5 × 10^5^ spores/g SH with the spores collected from PDA plates. The culture was then grown in a shaker (Innova 4080 Digital Incubator Shaker, New Brunswick Scientific) at 250 RPM and 25 °C. The samples were taken from each flask on 2, 4, 6, 7, 8, 9, 10, 11, 12, 13, 14, and 21 days for counting the spore concentrations. Each sample was counted three times. The profile of spore production in SmF was developed accordingly.

SSF experiments were made in 21 Erlenmeyer flasks (250 mL) containing 15 mL water, 10 g SH, and 10% of all other ingredients in the above SmF experiments. pH of the soluble nutrient medium was about 5.5. After autoclaving and cooling, the SSF systems were inoculated with 2.5 × 10^5^ spores/g SH, same as in the SmF flasks. Three flasks were taken as sacrificial samples each time on 2, 4, 6, 8, 10, 12, and 14 days for spore counting, following the same procedure for the SmF systems.

## SSF study for effects of culture conditions on spore production

SSF systems were prepared as described in the previous section, except for the following factors varied individually to study their effects on spore production (summarized in Table 2). (1) For the effect of spore inoculum level, spores were added at 1X, 2X, 3X, 4X, and 5X amounts, respectively, where 1X = 2.5 × 10^5^ spores/g SH. (2) For the effect of moisture, the water amount was varied from 5 to 25 mL in increments of 5 mL, corresponding to SH-to-water ratios of 1:0.5, 1:1, 1:1.5, 1:2, and 1:2.5, respectively. (3) For the effect of readily assimilable C-source (in addition to SH), 0, 0.05, 0.1, 0.3, and 0.4 g glucose were added in different systems. (4) For the effect of readily assimilable N-source (NH_4_)_2_SO_4_ supplementation, in addition to the 9–15.4% protein in SH [60], two batches of experiments were done. The first batch evaluated low-level supplementations, with 0, 0.005, 0.01, 0.03, and 0.04 g (NH_4_)_2_SO_4_, to test whether such supplementations could jump-start initial cell growth and enzyme synthesis (hypothetically helpful for extraction/hydrolysis of SH protein as N source). The second batch evaluated much higher levels of (NH_4_)_2_SO_4_: 0.354, 0.707, and 1.179 g, together with higher (fixed) levels of other mineral nutrients: 0.329 g KH_2_PO_4_, 0.127 g MgSO_4_.7H_2_O, 0.046 g CaCl_2_, and 0.001 g FeSO_4_. A control system without (NH_4_)_2_SO_4_ addition, but with addition of other mineral nutrients, was also included. The 4 systems corresponded approximately to 0% (control), 30%, 60%, and 100% N supplementation, where the 100% system had an added (NH_4_)_2_SO_4_ amount to support, hypothetically, complete consumption of SH carbohydrate for cell growth, estimated as follows: 10 g SH contains about 8.5 g carbohydrate and 1 g protein. Assuming 40% cell yield from carbohydrate, cells could grow to a maximum of 3.4 g (dry weight). Assuming 12% N content in cells, the total N required is 0.41 g. The 1 g protein in SH provides 0.16 g N (estimated with the common average of 16% N in protein). So, 0.25 (= 0.41–0.16) g N needs to be supplemented from 1.18 g (NH_4_)_2_SO_4_. (5) To observe the effect of adding enzymes or other metabolites produced in previous SSF to potentially jump-start cell growth, the 15 mL water typically added in SSF was replaced in a test system by 15 mL of an SSF extract prepared as follows: 80 mL sterile deionized water was added to a previously grown SSF flask. The flask contents were mixed for 10 min by a magnetic stir bar and then centrifuged to collect the supernatant extract (10 min, 10,000 rpm, Thermo Scientific™ Sorvall™ Legend™ X1R Centrifuge). (6) For the effect of spore inoculum preparation, SSF systems were inoculated with spores collected from either a PDA plate (control) or a previous SSF, at the same inoculation level of 2.5 × 10^5^ spores/g SH. (7) Water in the SSF flasks evaporated slowly over time, measured to lose (23.1 ± 0.4)% of water in 14 days. To test the effect of mid-process water addition and mixing (the latter needed to distribute the added water), two SSF systems were mixed with a sterile spatula at Day 7 with and without addition of 5 mL deionized water. Spore production results were compared with the control without mixing or water addition. (8) For the effect of SH particle size, SSF experiments were made with SH particles sieved into 3 size groups: small, 600–850 µm; medium, 850 µm–2 mm; and large, 2–5.6 mm. (9) For the effect of pH, two batches of SSF experiments were made. In one batch, the medium pH was adjusted to 5.5 (control) and 10, respectively, using 0.1 M NaOH before being added to SH and autoclaved, and the spore yields were measured and compared at Days 4, 8, and 14. In the other batch, the medium pH was adjusted to 5.5 (control), 9, and 11, respectively, and only the final spore yields at Day 14 were compared. (10) For the effect of NaCl addition, the SSF systems were compared without (control) and with addition of 1.5 g NaCl, corresponding to 10 g/L NaCl concentration in the initial aqueous medium.

**Table 2:** SSF factors tested for effects on *S. brevicaulis* spore production

Experiment	Tested factor	Tested factor levels	Details
1	Spore inoculum level	1X, 2X, 3X, 4X, 5X	1X = 2.5 × 10^5^ spores/g SH
2	Moisture	1:0.5, 1:1, 1:1.5, 1:2, 1:2.5	SH-to-water ratios
3	C-source (glucose) supplementation	0, 0.005, 0.01, 0.03, 0.04 g/g SH	Additional glucose to SH
4a	N-source ((NH_4_)_2_SO_4_) supplementation	0, 0.0005, 0.001, 0.003, 0.004 g/g SH	Low-level supplementations
4b		0, 0.0354, 0.0707, 0.1179 g/g SH	High-level supplementations, with also, per g SH: 0.0329 g KH_2_PO_4_, 0.0127 g MgSO_4_.7H_2_O, 0.0046 g CaCl_2_, 0.0001 g FeSO_4_
5	SSF extract addition	15 mL	Preparation: mixing 80 mL sterile water with a grown SSF, and centrifuging to collect supernatant extract
6	Spore source	PDA plate vs. previous SSF	Same inoculation level: 2.5 × 10^5^ spores/g SH
7	Mid-process mixing and water addition	0 or 5 mL water	Added and mixed at Day 7
8	SH particle size	Small (600–850 µm), Medium (850 µm–2 mm), Large (2 mm–5.6 mm)	Different SH particle sizes used in SSF
9a	Medium pH	5.5 (control) & 10	Spore yields measured at Days 4, 8, and 14
9b		5.5 (control), 9 & 11	Spore yields measured at Day 14
10	NaCl addition	With and without NaCl	0.15 g NaCl/g SH

### Spore collection and counting

Spore counting was all done in triplicate with diluted samples using a Double Neubauer Counting Chamber (VWR Catalog Number 15170-173) under a microscope at 100× magnification. For cultures grown on the 90-mm agar plates, the spore suspensions were prepared by adding 20 mL of 1 g/L Tween 80 (Fisher Scientific T164) and gently scraping the culture surface with a sterile wire loop. For SSF systems, the flask contents were added with 100 mL of the 1 g/L Tween solution and mixed for 5 min by a 38 mm × 8 mm octahedral stir bar at the maximum speed on a magnetic stir plate (Thermo Scientific, RT Basic Series). For spore counting, 0.5 mL of the mixture were diluted 40-fold with water, vortexed for 1 min, and then filtered through a mesh of 500 μm grids to remove large pieces of fungal biomass and unconsumed SH. For the SmF system, 1 mL culture broth was collected under mixing, diluted 40-fold with water, and then counted for spores.

### SEM analysis

For SEM analysis, samples taken from a SSF with large SH as substrate were air dried, mounted on carbon tapes, and analyzed using a Tescan LYRA3 XMU Scanning Electron Microscope (SEM). 3 kV electron energy was used with the BSE mode to capture the images at magnifications of 100X, 500X, and 1760X.

### Statistical analysis

Statistical analysis was done using the Minitab Software (version 20). ANOVA (Analysis of Variance) and Tukey's mean comparison tests at *p* < 0.05 were performed to determine the significant differences between treatments.

## Results and discussion

### Soy materials as substrate and effects of high pH, salt, and darkness on spore production

**Fig. 1:**
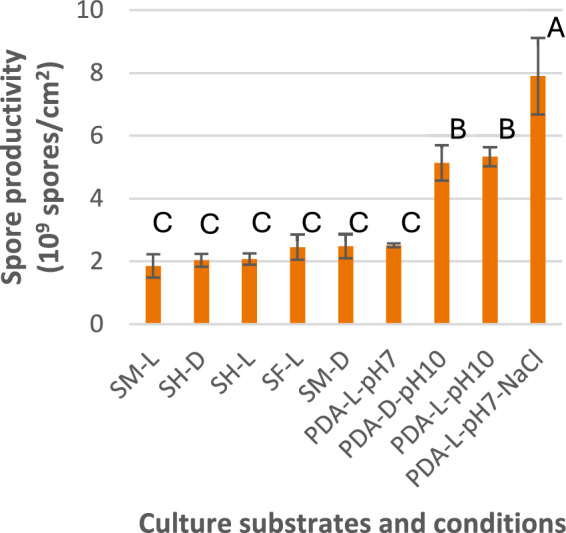
Effects of soy-based substrates and culture conditions: pH, NaCl addition, and light (L) vs. darkness (D), on spore production of *S. brevicaulis* grown on agar plates; systems arranged in the order of increasing average spore productivity from left to right, showing enhanced production in systems with NaCl addition (Group A according to Tukey’s test) and the higher pH 10 (Group B) but no significant effects from the other factors (Group C)

For the nine systems tested on agar plates (Table 1), the number of spores produced per cm^2^ of the agar plate surface area after 14 days of cultivation are compared in Fig. 1. The systems that had no significant differences in spore production, according to Tukey’s pairwise comparison, are labeled with the same letter (A, B, or C).

For the soy-based substrate effect, the cells grew as very thin layers on the agar plates with SM and SF, and sporulation appeared to occur only in the central region of the colonies, whereas on plates with SH, spores formed in the whole colonies. Despite these visual differences, the total numbers of spores produced after 14 days from the SH, SF, and SM systems were not statistically different (see the results in Fig. 1 for SH-L, SF-L and SM-L systems). Therefore, both SH and SF can possibly serve as solid substrate for SSF. However, SH is much cheaper at about $100/ton while SF is about $400/ton. In addition, SH is easier to work with for SSF, serving both as the substrate and the rigid support, requiring no inert support/carrier, whereas SM and SF need inert carrier or support to create void space for heat and oxygen transfers.

For the effects of culture conditions/factors, the light (L) and dark (D) conditions did not have significant effects in all three pairs of Tukey’s comparison: Pair 1 (SH-L and SH-D) and Pair 2 (SM-L and SM-D), with SH and SM as substrate, respectively, were all in the same Group C (Fig. 1), and Pair 3 (PDA pH10-L and PDA pH10-D), with PDA as substrate and initial medium pH 10, were both in Group B (Fig. 1). On the other hand, high initial pH (10) and, particularly, high salt (15 g/L) conditions were effective in raising spore production. Both above pH 10 systems were in Group B, giving 2.3-fold higher spore production than the Group C growing with an initial pH of 7 (including the control on PDA, i.e., the PDA pH7-L system). Only the 15 g/L NaCl system (PDA pH7-NaCl-L) was in the most productive Group A, producing 3.5-fold more spores than Group C growing at pH 7 and without NaCl addition. Hypothetically, both the high pH and salt (osmolality) created stressed conditions that triggered more extensive sporulation.

### Comparison of *S. brevicaulis* spore production in SSF and SmF

**Fig. 2:**
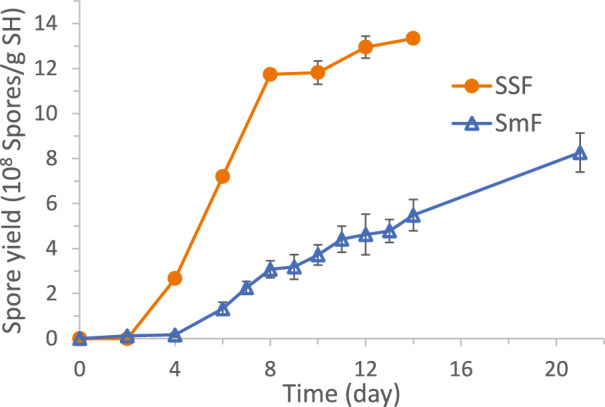
Comparison of spore production profiles in SSF and SmF using SH as substrate

The time profiles of *S. brevicaulis* spore yields obtained in SSF and SmF using SH as substrate (without high initial pH or salt addition) are compared in Fig. 2. The SSF system did not produce spores in the first two days, produced spores almost linearly during Day 4 to Day 8, and started to plateau afterwards. The spore yield (spores/g SH) during Day 2 to Day 8 followed the best-fit linear equation: (Spore yield) = -4.54 × 10^8^ + 1.99 × 10^8^ (Day) (R^2^ = 0.98). The maximum spore yield reached on Day 14 was (1.33 ± 0.02) × 10^9^ spores/g SH. The SmF system did not produce spores for about 4 days and produced spores almost linearly afterwards. The spore production during Day 6 to Day 21 can be described by the equation: (Spore yield) = -7.62 × 10^7^ + 4.39 × 10^7^ (Day) (*R*^2^ = 0.98). The daily spore productivity in SmF, i.e., 4.39 × 10^7^ spores/(g SH-day), was only about 22% of that in SSF, i.e., 1.99 × 10^8^ spores/(g SH-day). The spore yield of the SmF was (5.49 ± 0.07) × 10^8^ spores/g SH on Day 14 and (8.27 ± 0.09) × 10^8^ spores/g SH on Day 21, much lower than the (1.17 ± 0.01) × 10^9^ spores/g SH reached in the SSF by Day 8. It can be concluded that SSF offered substantially higher *S. brevicaulis* spore productivity than the SmF. Feng et al. also reported higher productivity of *V. lecanii* spores by SSF than SmF, although the time profile of spore production was only shown for a 12-day SmF [19]. Moreover, the SSF-produced aerial spores of *Trichoderma harzianum* were reported to be more tolerant to stress and UV than those produced by SmF [61], suggesting benefit of spore production by SSF over SmF. SSF consumes less energy [62] and produces less effluent, representing a cleaner way of producing bioproducts [63]. Accordingly, SSF was chosen for further study in more detail.

### Effects of culture conditions on spore production by SSF

#### Spore inoculum level

**Fig. 3:**
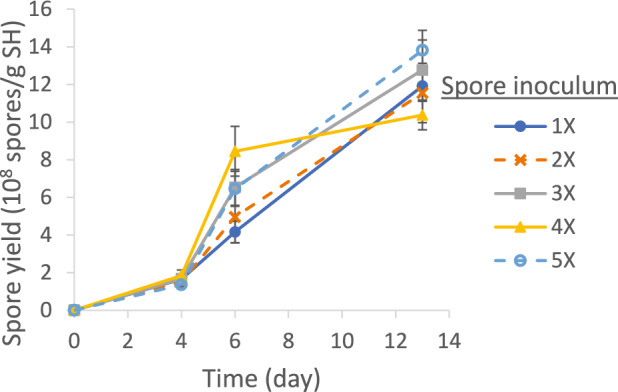
Effect of spore inoculation levels (1X = 2.5 × 10^5^ spores/g SH) on SSF spore production yield

As shown in Fig. 3, the systems inoculated with different spore levels (1X, 2X, 3X, 4X, and 5X, with 1X = 2.5 × 10^5^ spores/g SH) followed similar spore production patterns. On Day 6, the spore production was higher in the systems with higher inoculum levels (3X, 4X, and 5X), presumably due to higher initial concentrations of germinating cells; nonetheless, the up to 70% difference was far from being proportional to the inoculum level (up to 500%). In addition, the difference in the final spore production on Day 13 further diminished (all in the same grouping by the Tukey method at 95% confidence). Viccini et al. also observed no statistical difference in the production of *C. rosea* spores by SSF with different inoculum levels [36]. Accordingly, 1X spore inoculation was used in all subsequent experiments.

#### Moisture content

**Fig. 4:**
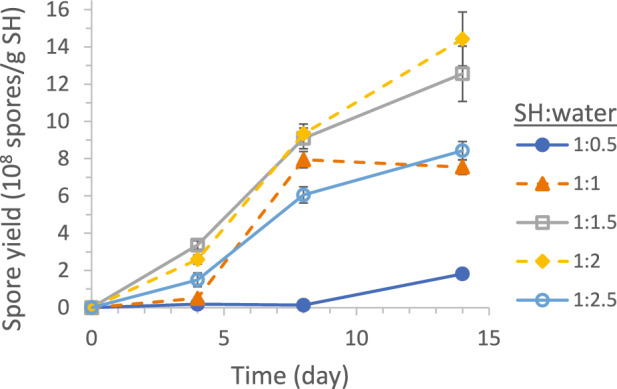
Effect of SH-to-water ratio on SSF spore production yield

Spore production profiles in systems with different SH-to-water ratios of 1:0.5, 1:1, 1:1.5, 1:2, and 1:2.5 are shown in Fig. 4. At SH:water = 1:0.5, the moisture content was insufficient to support the biological activities. Water is not only an essential component of live cells (making up about 90% of wet cell weight) but also the medium that enables extracellular transfer of soluble nutrients and enzymes, which hydrolyze SH to assimilable compounds like sugars and amino acids. Spore production first improved significantly with increasing moisture content and maximized at SH:water = 1:1.5 to 1:2, but then declined at an even higher moisture content (SH:water = 1:2.5). Similar finding was reported by Dorta-Vásquez et al. for their SSF production of *Trichoderma asperellum* spores in SSF with papermaking sludge as feedstock, where they observed the spore producitivity to peak at water contents of 60% and 70% but decline at 80% and 90% [39]. Note that water contents of 60%, 70%, 80%, and 90% correspond to SH:water ratios of 1:1.5, 1:2.4, 1:4, and 1:9, respectively. Our SSF of *S. brevicaulis* on SH was found to be more sensitive to high water contents, with spore production already declining at water content of 70%. Exact reasons for this higher sensitivity are unclear but increasing water content beyond the saturation level absorbable by the solid substrate would reduce interparticle airspace and impede oxygen transfer into the SSF bed. The finding is also consistent with the lower spore production in SmF (with plenty of water) than SSF reported in the previous section, although the controlling mechanism(s) could be different in the two fermentation types.

#### Glucose supplementation

**Fig. 5:**
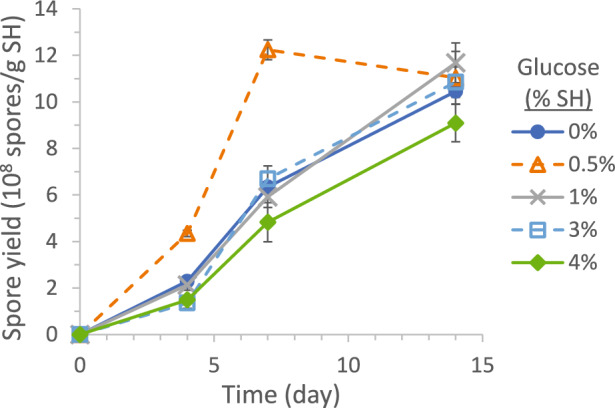
Effect of sugar addition, in wt% of SH, on SSF spore production yield

Figure 5 shows the spore production profiles in systems containing 0, 0.05, 0.1, 0.3, and 0.4 g glucose (per 10 g SH and 15 mL water, i.e., equivalent to 0%, 0.5%, 1%, 3%, and 4% of SH weight). A low-level (0.5%) glucose addition increased the early spore production measured at Day 4 and Day 7 but not the final production at Day 14. The positive effect diminished at 1% and 3% glucose, and the effect even turned negative at 4% glucose. The fungus needs to synthesize enzymes and hydrolyze SH to assimilable sugars and amino acids. These activities take time and are probably the rate-limiting factors for cell growth. The systems all had 0.02 g (NH_4_)_2_SO_4_ as a ready N source for cell growth and enzyme synthesis. It appeared that the initial growth stimulated by the low-level 0.5% glucose addition could be sustained by subsequent SH hydrolysis, resulting in the faster early-stage spore production, but higher-level glucose additions might exhaust all (NH_4_)_2_SO_4_ for cell growth without adequate enzyme synthesis to sustain the growth. Further, a similar final spore yields at Day 14 (all systems in the same grouping by the Tukey method at 95% confidence) suggested the maximal spore yields in these SSF systems were not limited by the C-source amounts available and, therefore, were not significantly affected by glucose additions.

#### N-source supplementation

**Fig. 6:**
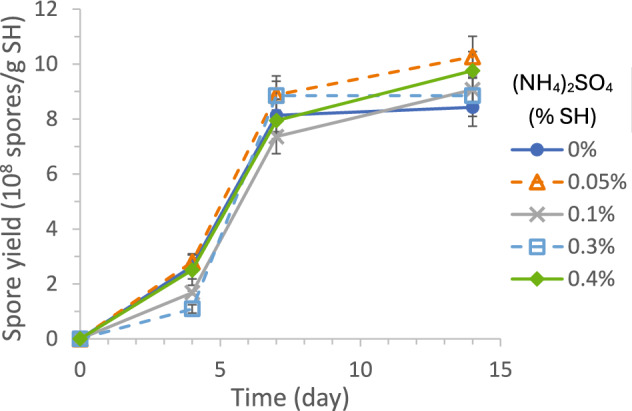
Effect of low levels of N-source ((NH_4_)_2_SO_4_) supplementation, in wt% of SH, on SSF spore production yield

SH has about 9–15.4% protein [58, 60], but its utilization by cells requires SH and protein hydrolysis. (NH_4_)_2_SO_4_ was first added at 0, 0.005, 0.01, 0.03, and 0.04 g, respectively, to investigate if low-level supplementations of a readily assimilable N-source could jump-start cell growth and enzyme synthesis to affect spore production. As shown by the results in Fig. 6, there was no clear evidence to support the above hypothesis. It should be noted that the above amounts corresponded to 0%, 0.05%, 0.1%, 0.3%, and 0.4% of SH (by weight), respectively, which were much lower than the 9–15.4% protein in SH. Therefore, they were not expected to significantly affect the final cell growth at Day 14, even if the cell growth was N-limited. The findings further support the advantageous use of SH as the SSF substrate, requiring minimal or negligible supplementation of C and N sources as reported in Sect. "Glucose supplementation" and this section.

**Fig. 7:**
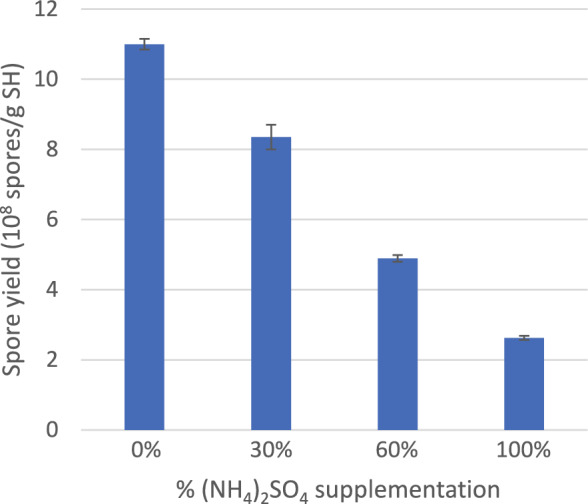
Effect of high levels of (NH_4_)_2_SO_4_ supplementation on SSF spore production yield

To see if higher-level supplementation of N and other mineral nutrients would improve cell growth and spore production, the second batch of experiments compared 0%, 30%, 60%, and 100% N supplementation, where the 100% system was added with 1.179 g (NH_4_)_2_SO_4_ to support, hypothetically, complete consumption of SH carbohydrate for cell growth (details described in Materials and Methods). This addition was much higher, about 30- to 236-fold, than those above (Fig. 6). These high-level N nutrient supplementations clearly increased cell growth (visually observable by the increasing coverage of SH particles with white mycelia) but either delayed or prevented sporulation (visually observable by the color change from white mycelia to brown spores). The final spore yields from these four systems are shown in Fig. 7. With increasing N supplementation, the sporulation delay increased and the spore yield decreased.

N-source depletion is a known condition to trigger/promote sporulation [55]. The delayed sporulation with increasing N supplementation is reasonable. However, the lower spore yields, even at 30% N supplementation, were not anticipated. As described in Materials and Methods, the 100% N supplementation was assumed to make the nutrients balanced in carbohydrate and N-source (and other mineral nutrients), i.e., to enable complete consumption of SH carbohydrate for cell growth (at an assumed 40% cell yield from SH carbohydrate). Because of the N available from about 10% protein present in SH, the 0%, 30%, and 60% (NH_4_)_2_SO_4_ supplementations were estimated to support up to 39%, 58%, and 76%, respectively, consumption of SH carbohydrate for cell growth (calculations not shown). The finding of lower spore yield at 30% (NH_4_)_2_SO_4_ supplementation probably indicates that lower than 58% SH carbohydrate was readily accessible or hydrolyzable for cell consumption so that with 30% or more (NH_4_)_2_SO_4_ supplementation, cells would run into energy-source limitation before the N-source depletion occurred to trigger/promote spore production.

#### SSF extract and spore source (SSF vs. PDA)

**Fig. 8:**
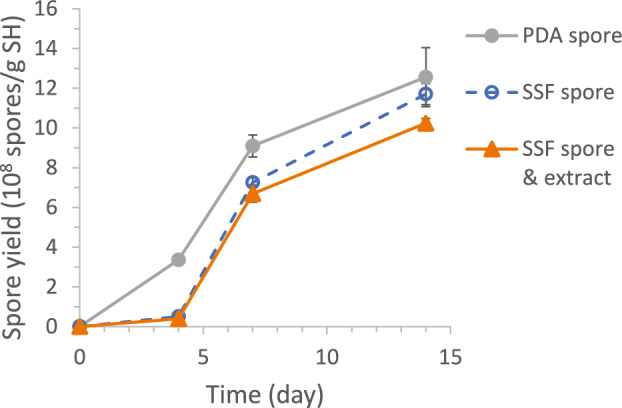
Comparison of SSF spore production profiles for systems having SSF spores as inoculum, with and without SSF extract (in place of water for SSF medium preparation), against the control inoculated with PDA spores

As shown in Fig. 8, the SSF extract, assumed to contain enzymes and other metabolites, did not show significant effect in jump-starting early cell growth and spore production. The two systems inoculated with SSF spores had slower spore production at Days 4 and 7, compared with the system with spores from PDA plates. Nonetheless, the final spore productivity at Day 14 was similar in all systems (in the same grouping by the Tukey method at 95% confidence). SSF spores were, therefore, suitable as the inoculum for subsequent SSF batches.

#### Mid-process mixing and water addition

The final (Day 14) spore yields, in (number of spores/g SH), for the three tested systems were: control (no mixing or water addition)—(1.10 ± 0.02) × 10^9^, mixing at Day 7 (no water addition)—(1.08 ± 0.09) × 10^9^, and mixing and 5-mL water addition at Day 7—(0.99 ± 0.04) × 10^9^, respectively. The statistical analysis by Turkey’s pairwise comparison did not find significant difference among the three systems. Mid-process mixing and water addition did not affect the final spore production under the tested conditions.

#### Soy hull size

**Fig. 9:**
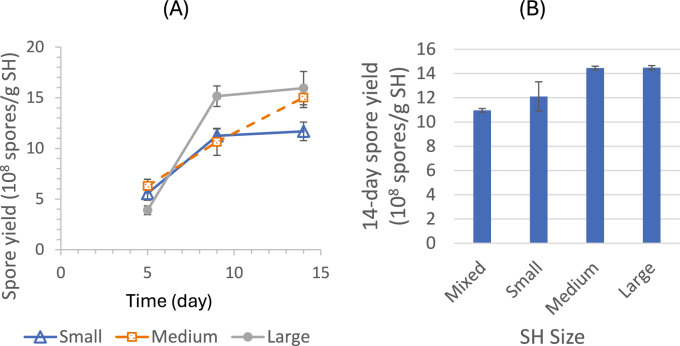
Effects of SH particle size on spore yields compared in two batches of SSF experiments

The effect of SH particle size on SSF spore production was studied in two batches of experiments. The SH particles were sieved into four size groups: fine, < 600 μm; small, 600–850 µm; medium, 850 µm–2 mm; and large, 2–5.6 mm. The original sample with mixed SH particles was determined to have 38% (w/w) fine particles, 14% small particles, 41% medium particles, and 7% large particles. Figure 9A shows the spore production profiles obtained in one batch of SSF experiments comparing 3 SH-size groups: small, medium, and large. Figure 9B shows the final (14-day) spore yields obtained in the other batch of SSF experiments, where four systems with mixed/original, small, medium, and large SH particles, respectively, were evaluated. The results from both batches of experiments indicated that after 14 days, the SSF spore yields from large and medium SH particles were comparable while the yields from small and mixed SH particles were significantly lower.

**Fig. 10:**
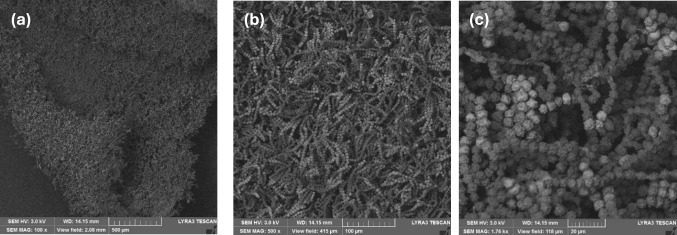
SEM pictures of SSF grown fungal spores on SH particles after 14 days, showing **a** a SH particle covered with spores (100X), **b** spores formed in chains (500X), and **c** spore shape and rough, spiky surface (1760X)

One reason for the better spore yields from larger SH particles was they created larger void spaces to allow faster and more homogeneous oxygen transfer into the bed of solid substrate. For the same amount of SH, the initial bed volume was estimated at 70 cm^3^ for the large-sized SH, 40 cm^3^ for the medium-sized SH, and 30 cm^3^ for the small- and mixed-sized SH. Further, as shown by the SEM pictures in Fig. 10, the chains of spores were formed as layers that attached on and covered all available SH surfaces. For dry SH particles, the surface area per g SH is expected to be larger with smaller particles. But, when water was added, the small SH particles aggregated together, reducing the surface area available for the inoculated spores to attach. This aggregation decreased significantly with increasing SH particle size and the associated curvature. Accordingly, the larger SH particles not only created more void space for better oxygen transfer but also offered larger SH surface area for inoculated spores to attach, germinated cells to grow on, and, subsequently, spore layers to form.

##### High pH

**Fig. 11:**
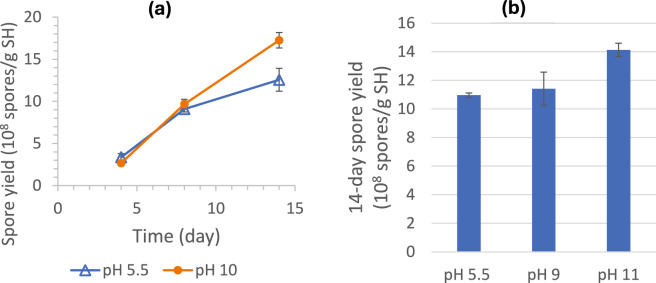
Effect of pH on SSF spore yield: **a** time profiles of spore yields obtained in SSF with initial medium pH of 5.5 and 10, showing comparable spore production in up to 8 days but higher production after 14 days in the SSF with high medium pH; **b** final spore yields after 14 days of SSF with initial medium pH of 5.5, 9, and 11 (three flasks at each pH), showing a clearly higher spore yield at pH 11

The positive effects of high pH on *S. brevicaulis* spore production were shown by both experiments with Petri-dish agar (PDA) plates (as shown in Fig. 1) and SSF flasks. Results of the two batches of SSF experiments are shown in Fig. 11. The time profiles in Fig. 11a showed that the systems at pH 5.5 and 10 had similar spore yields at Days 4 and 8 but the pH 10 system produced significantly more spores at Day 14 (*p* = 0.015). The findings suggested the higher pH was not more favorable for cell growth but could trigger more spore production in the later stage. The results in Fig. 11b showed that at Day 14, the spore yields from the pH 5.5 and 9 systems were not statistically different (*p* = 0.614) but the spore yield from the pH 11 system was significantly higher (*p* = 0.010). Both batches of experiments indicated more spore production in SSF with high initial medium pH, and the beneficial high-pH effect was significant only when the initial pH was higher than 9 (apparent at pH 10 and 11). One possible reason is that high pH represents a stressed condition, which triggers/stimulates more sporulation. This possibility is high when considered together with the high spore production in high-salt media, another potentially stressed condition, as described in the next section. High pH might have also physically and/or chemically affected the SH substrate, particularly during autoclaving, making SH more amenable to hydrolysis and fungal consumption.

##### High salt concentration

A SSF system was made with 10 g/L NaCl in the aqueous medium added, together with the salt-free control. The 14-day spore yields were (1.09 ± 0.08) × 10^9^ spores/g SH in the salt-free control and (1.3 ± 0.2) × 10^9^ spores/g SH in the 10 g/L NaCl system. While the average spore yield from the salt-added system was higher, the two systems did not have significant difference statistically (*p* = 0.17) because of the large standard deviation found in the 10 g/L NaCl system. On the other hand, as shown in Fig. 1, the addition of 15 g/L NaCl to the culture medium (PDA) significantly increased the spore production: from (2.5 ± 0.1) × 10^9^ spores/(cm^2^ plate area) in the control without NaCl addition to (7.9 ± 1.2) × 10^9^ spores/(cm^2^ plate area) in the NaCl added system. The results suggested that a high salt concentration can significantly improve the SSF spore production and the optimal salt concentration to add is higher than 10 g/L, with 15 g/L (roughly one half of the salinity level of sea water) giving a much more significant improvement.

### Comparison with fungal spore yields reported in the literature

The yield of *S. brevicaulis* spores by SSF in this work (which had not been reported before) is compared in Table 3 with the spores yields of other fungi reported in the literature. The spore yield obtained in this work compared well with others, particularly when the larger size of *S. brevicaulis* spores was considered.

**Table 3:** Comparison of fungal spore yields by SSF

Species	Spore size (μm)	Substrate	Max spore yield (per g substrate)	References
*Beauveria bassiana*	2–3 [18]	Rice husk + nutrients (dextrose, NaNO_3_, KH_2_PO_4_, MgSO_4_)	4.3 × 10^8^ to 1.8 × 10^9^ (w/ and w/o added nutrients)	[32, 64]
*Verticillium lecanii*	5–6.1 [19]	Sugarcane bagasse	1 × 10^10^	[65]
*V. lecanii*		Sugarcane bagasse + nutrients (polyurethane foam + corn flour, yeast extract, KH_2_PO_4_, K_2_HPO_4_, MgSO_4_)	1.1 × 10^10^	[33]
*Coniothyrium minitans*	4–5 [66]	Oats	5 × 10^9^	[35]
*C. minitans*		Wheat bran + nutrients (glucose, (NH_4_)_2_SO_4_, K_2_HPO_4_, CaCl_2_·2H_2_O, MgCl_2_·6H_2_O, FeSO_4_·7H_2_O)	1.5 × 10^10^	[34]
*Clonostachys rosea*	2.9 [20]	White rice grain	3.4 × 10^9^	[36]
*Trichoderma guizhouense*	2.8–2.9 [67]	Stevia residue + amino acids	7 × 10^9^	[37]
*Trichoderma*^a^	2–4 [68]	Mixture of sugarcane bagasse, wheat bran, chitin, potato flour, and olive oil	1 × 10^9^	[38]
*Trichoderma asperellum*	2.9 [69]	Papermaking sludge + fertilizers (Nutrisol P®, Nutrisol K®)	2.4 × 10^9^	[39]
*S. brevicaulis*	5	Soy hull	1.7 × 10^9^	This work

^a^*T. longibrachiatum, T. harzianum, T. yunnanense, T. asperellum* (T2-10 and T2-31), and *Trichoderma sp.* from Department of Agricultural Parasitology of UAAAN (Universidad Autónoma Agraria Antonio Narro, Coahuila, Mexico)

### Novelty, limitations, and future work

The development of SHC has so far been focused on using bacterial endospores, which require higher costs for spore production and collection. Fungi are more effective in producing extracellular enzymes to grow on cheap and sustainable solid substrate or waste, and as found in this study, can produce spores by SSF with higher productivity than by SmF. Also, fungal spores are much larger than bacterial endospores and thus easier to collect. Overall, using fungal spores can significantly lower the spore cost when used in SHC. Other novelties of this work include the use of stress factors (high pH and NaCl concentration) to induce spore production and the use of large, curved SH particles as substrate to create high porosity and large pores in the SSF bed. Further, production of *S. brevicaulis* spores has never been studied/reported before.

SSF represents a cultivation technique where fungi are grown on a solid substrate without the presence of free-flowing liquid. It mimics their natural habitats and leverages low-cost agricultural residues or waste products as substrates, thereby presenting an economically viable and cleaner option to minimize waste generation and energy consumption. For spore production, the SSF boasts advantages such as reduced contamination risk and potentially higher spore yields than SmF. However, it presents challenges in precisely controlling environmental parameters (e.g., temperature, oxygen transfer and pH) and can be more difficult to scale up. The findings of this study indicated that the use of stressed conditions such as high pH and NaCl can significantly enhance spore productivity. Such conditions potentially reduced the fungal metabolic rate (compared to that under optimal growth) and, thus, metabolic heat release and respiration, lowering the challenges in controlling temperature and oxygen transfer. The favorable spore production found when using larger SH pieces, which creates larger porosity and pores, further helps with the SSF scaleup. Nonetheless, future work on scaleup of the spore production by soyhull-based SSF is necessary. Future research is also warranted to compare properties like viability, storability, and tolerance to concrete-relevant environments, for the *S. brevicaulis* spores produced by SSF and SmF.

## Conclusion

Self-healing concrete (SHC) offers a more sustainable approach to infrastructure maintenance by leveraging the biomineralization ability of microorganisms. A critical step toward enabling this technology is the development of a cleaner and more cost-effective method for producing the large quantities of spores required for SHC applications. This study demonstrated that solid-state fermentation (SSF) using soy hulls (SH) as substrate is a significantly more effective method for producing *S. brevicaulis* spores than submerged fermentation (SmF). SSF resulted in faster production rates, higher yields, and minimal wastewater. In SSF, SH functioned both as a nutrient source and as a structural support that created voids, facilitating improved oxygen and heat transfers.

Several process variables were investigated. Among the factors tested, the following had no significant impact on final spore yields: light versus dark conditions, inoculation levels (1X–5X, with 1X = 2.5 × 10^5^ spores/g SH), glucose supplementation (0–4% of SH weight), low-level (NH₄)₂SO₄ supplementation (0%–0.4%), spore source (PDA vs. SSF), addition of SSF extract (containing enzymes and other metabolites), and mid-process mixing with or without water addition. In contrast, other factors showed clear effects. High-level (NH₄)₂SO₄ supplementation (3.5–11.8%), intended to promote more complete SH consumption, resulted in progressively lower spore yields with increasing supplementation. Moisture content had a strong influence: minimal sporulation occurred at 33.3% (SH:water = 1:0.5), yields improved at 50%, peaked at 60–67%, and declined at 71.4%, likely due to reduced oxygen transfer as excess water filled interstitial spaces. SH particle size also affected outcomes: large (2–5.6 mm) and medium (850 µm–2 mm) particles supported higher yields than small (600–850 µm) or mixed sizes, likely because larger particles created greater bed porosity and enhanced oxygen transfer. In addition, high initial medium pH (10 and 11, but not 9) and elevated NaCl concentrations (15 g/L, but not 10 g/L) substantially increased spore production, likely due to stress-induced sporulation.

This is the first reported study on *S. brevicaulis* spore production. The highest yield achieved was 1.7 × 10⁹ spores/g SH after 14 days, with an average production rate of 1.2 × 10⁸ spores/(g SH-day). The highest observed rate during the active sporulation phase was 2.0 × 10⁸ spores/(g SH-day). In addition to improved spore productivity, SSF offers another important advantage: spores are produced in a concentrated, relatively dry form that is easier to collect and store. In contrast, spores produced by SmF are suspended in large volumes of water, requiring energy-intensive processes such as centrifugation to collect and drying or refrigeration to maintain viability. Overall, this study significantly advances the technical and economic feasibility of producing *S. brevicaulis* spores for applications such as their use as a bioagent in SHC.
